# Extracellular Superoxide Dismutase Attenuates Hepatic Oxidative Stress in Nonalcoholic Fatty Liver Disease through the Adenosine Monophosphate-Activated Protein Kinase Activation

**DOI:** 10.3390/antiox12122040

**Published:** 2023-11-24

**Authors:** Heechul Nam, Ji Hee Lim, Tae Woo Kim, Eun Nim Kim, Sae-Jong Oum, Si Hyun Bae, Cheol Whee Park

**Affiliations:** 1 Division of Hepatology, Department of Internal Medicine, Catholic University of Korea, Seoul 06591, Republic of Korea; hcnam128@catholic.ac.kr; 2 Division of Nephrology, Department of Internal Medicine, Catholic University of Korea, Seoul 06591, Republic of Korea; didsuai@hanmail.net (J.H.L.); tomcat0720@gmail.com (T.W.K.); kun0512@catholic.ac.kr (E.N.K.); sjoum1016@gmail.com (S.-J.O.); 3 Department of Medicine, School of Medicine, St. George’s University, St. George 11739, Grenada; 4 Institute for Aging and Metabolic Diseases, Catholic University of Korea, Seoul 06591, Republic of Korea

**Keywords:** adenosine monophosphate-activated protein kinase, extracellular superoxide dismutase, nonalcoholic fatty liver disease

## Abstract

Oxidative stress is key in type 2 diabetes-associated nonalcoholic fatty liver disease (NAFLD). We explored whether extracellular superoxide dismutase (EC-SOD) activates adenosine monophosphate-activated protein kinase (AMPK) to enhance antioxidant synthesis and lipid metabolism in NAFLD. Human recombinant EC-SOD (hEC-SOD) was administered to 8-week-old male C57BLKS/J *db*/*db* mice through intraperitoneal injection once a week for 8 weeks. Target molecules involved in oxidative stress and lipid metabolism were investigated. hEC-SOD improved insulin resistance and systemic and hepatic oxidative stress characterized by increases in urinary 8-hydroxy-deoxyguanosine and 8-isoprostane levels in *db*/*db* mice and a decrease in DHE expression in the liver, respectively. Hepatic SOD3 expression in *db*/*db* mice was reversed by hEC-SOD, which improved hepatic steatosis, inflammation with M2 polarization, apoptosis, autophagy, fibrosis and lipid metabolism in *db*/*db* mice, as reflected by the changes in serum and hepatic markers, monocyte chemoattractant protein-1, tumor necrosis factor-α, TUNEL-positive cells, Bcl-2/BAX ratio, beclin1 and LC3-II/LC3-1. At the molecular level, hEC-SOD increased phosphorylated-AMPK related to CaMKKß, activation of peroxisome proliferative-activated receptor-gamma coactivator (PGC)-1α and dephosphorylation of forkhead box O (FoxO)1 and their subsequent downstream signaling. In HepG2Cs cells using *AMPKα1* and *AMPKα2* siRNA, hEC-SOD demonstrated a protective effect via the direct activation of both AMPK-PGC-1α and AMPK-FoxO1. EC-SOD might be a potential therapeutic agent for NAFLD through the activation of AMPK-PGC-1α and AMPK-FoxO1 signaling in hepatocytes, which modulates lipid metabolism, leading to anti-inflammatory, antioxidative and antiapoptotic effects and improving autophagy in the liver.

## 1. Introduction

Nonalcoholic fatty liver disease (NAFLD) is characterized by the accumulation of fat in the liver and is intimately intertwined with metabolic syndromes [1]. The “two-hit” model partially explains the varying progression to more severe hepatic inflammation and fibrosis in patients with hepatic steatosis [2]. The first “hit” occurs when insulin resistance (IR) leads to the accumulation of fat in the liver cells. The second “hit” is triggered by the production of reactive oxygen species (ROS) due to the excess fat in the liver. The overproduction of ROS, exceeding the capacity of the antioxidant system to neutralize them, induces oxidative stress. This oxidative stress activates a cascade of inflammatory cytokines that contribute to the progression of necroinflammation and fibrosis. Furthermore, oxidative stress disrupts the delicate balance between antioxidant defenses and pro-oxidant factors in the liver, further promoting the evolution of NAFLD [3]. The molecular mechanism of lipotoxicity is characterized by endoplasmic reticulum stress, inflammation, impaired autophagy and excessive oxidative stress related to mitochondrial dysfunction [4]. Among these, oxidative stress is thought to be the most important causative agent of lipotoxicity-induced organ damage [5,6].

Therefore, enzymatic antioxidants play a crucial role in providing effective protection against oxidative damage due to their capability to break down ROS. Among these enzymatic antioxidants, superoxide dismutase (SOD) holds particular importance for living cells, as the majority of ROS originate from superoxide [7]. SOD catalyzes the conversion of superoxide into oxygen and hydrogen peroxide. Mammals possess three distinct forms of SOD, each characterized by its metal ions and subcellular localizations. Copper–zinc SOD (SOD1) resides in the cytosol, manganese SOD (SOD2) is located in the mitochondria, and extracellular SOD (EC-SOD or SOD3) primarily occupies extracellular spaces, playing a unique role in maintaining tissue homeostasis in the extracellular environment [8,9]. In previous studies involving patients with type 2 diabetes and metabolic syndrome, it has been reported that serum EC-SOD activity is negatively correlated with insulin resistance [10,11]. However, the role of EC-SOD as a potential therapeutic target for NAFLD has yet to be elucidated.

Adenosine monophosphate-activated protein kinase (AMPK) is a metabolic sensor and regulator of systemic energy balance [12]. The activation of AMPK and its subsequent downstream signaling pathway of lipid metabolism reduces the accumulation of triglycerides, fatty acids and cholesterol in the liver and attenuates the development of NAFLD [13]. AMPK also plays a central role in controlling energy metabolism by modulating a plethora of other downstream targets, including proliferator-activated receptor gamma coactivator-1α (PGC-1α), forkhead box O transcription factor (FoxO), the mammalian target of rapamycin and silent information regulator 1 [14,15]. PGC-1α is widely recognized as a transcriptional coactivator, being primarily responsible for regulating mitochondrial biogenesis and oxidative metabolism. As another additional transcriptional factor, FoxO exerts its influence on an array of cellular processes, including stress resistance, oxidative stress and cell survival [16,17].

We previously reported that several AMPK activators mitigate the severity of diabetic kidney disease by improving oxidative stress through the activation of AMPK and its downstream signaling pathways [18,19,20]. However, the protective effects of EC-SOD via the activation of AMPK in NAFLD remain poorly understood. We propose that human recombinant EC-SOD (hEC-SOD) has the potential to alleviate metabolic-dysfunction-induced oxidative stress, inflammation and apoptosis through the activation of AMPK and associated downstream targets.

## 2. Materials and Methods

### 2.1. Preparation of Recombinant EC-SOD

Recombinant EC-SOD was synthesized following previously described methods [21]. Briefly, 293 cells underwent transient transfection using the SOD3 construct for 48 h. Post-transfection, the supernatant was harvested and underwent purification via Ni-NTA agarose column (Qiagen, Valencia, CA, USA), which was then followed by dialysis. The activity of the purified SOD3 was quantified using an SOD assay kit (Dojindo, Sunnyvale, CA, USA). Prior to being administered to mice or used in vitro, SOD3 was filtered to remove any endotoxin.

### 2.2. Animals and Treatment

Male C57BLKS/J *db*/*m* and *db*/*db* mice, aged eight weeks, were sourced from Jackson Laboratories (Bar Harbor, ME, USA). These genetically modified mouse models are widely used in biomedical research, particularly for studying obesity, diabetes and related metabolic disorders. Metabolically, they display insulin resistance, hyperglycemia and dyslipidemia, mirroring human NAFLD. Their liver pathology, including steatosis, inflammation and fibrosis, closely resembles the progression in humans from fatty liver to more severe stages like NASH and cirrhosis. They were allocated into four groups and maintained on a standard chow diet. *Db*/*db* and age-gender-matched *db*/*m* mice (each group n = 8) received weekly intraperitoneal injections of hEC-SOD (3500 U/(kg/day), 120 µL) for eight weeks. Concurrently, control groups of *db*/*db* and *db*/*m* mice (each group n = 6) received equivalent doses of saline. At 16 weeks, the mice were anesthetized with 30 mg/kg tiletamine/zolazepam (Zoletil; Virbac, Carros, France) and 10 mg/kg xylazine hydrochloride (Rompun; Bayer, Leverkusen, Germany) and then euthanized. Liver tissues were promptly excised and preserved in 10% buffered formalin. Blood samples were drawn from the left ventricle, with plasma stored at −70 °C.

### 2.3. Blood and Urine Parameters

Blood samples from the animals were collected following overnight fasting. Fasting blood glucose levels were assessed using an Accu-check meter (Roche Diagnostics, St Louis, MO, USA). Glycosylated hemoglobin (HbA1c) levels were determined using an autoanalyzer (Bayer Corporation, Elkhart, IN, USA). Aspartate aminotransferase and alanine aminotransferase were measured using absorbance assays. Plasma insulin concentrations were measured using an RIA (Alpco, Salem, NH, USA). Plasma insulin levels were determined through a radioimmunoassay (RIA) by Alpco. The homeostatic model assessment for insulin resistance (HOMA_IR_) was calculated using the formula fasting glucose (mmol/L) × fasting insulin (mU/L)/22.5. Oxidative DNA damage and lipid peroxidation were evaluated by measuring 24 h urinary levels of 8-hydroxy-deoxyguanosine (8-OH-dG; OXIS Health Products, Portland, OR, USA) and 8-epi-prostaglandin F2α (isoprostane; OXIS Health Products), respectively.

### 2.4. Histological Assessment

Histological examination involved using 5 μm thick sections of paraffin-embedded tissue, which were subsequently stained with hematoxylin and eosin and Masson’s trichrome. We also performed immunohistochemistry to transform growth factor-β (TGF-β)1 (R&D Systems, Minneapolis, MN, USA), examined it under bright-field illumination (Olympus Optical, Shinjuku-ku, Tokyo, Japan, Olympus BX50) using a 40× objective lens and analyzed it using ImageJ (version 1.53). For immunofluorescence staining, liver sections were incubated with antitumor necrosis factor-α (TNF-α) (Abcam, Cambridge, UK) and anti-perilipin-2 (Abcam), followed by incubation with fluorescent-dye-conjugated secondary antibodies (Alexa Fluor 555-conjugated anti-mouse IgG, Thermo Fisher Scientific, Waltham, MA, USA). To determine the percentage of apoptotic cells, a terminal deoxynucleotidyl transferase-mediated dUTP nick end-labeling (TUNEL) assay was conducted, employing the ApopTag^®^ kit Fluorescein In Situ Apoptosis Detection Kit (Millipore, Burlington, MA, USA). Additionally, frozen liver tissues were stained with Oil Red O (Sigma Aldrich, St. Louis, MO, USA), and ROS accumulation was determined via a DHE Assay (Sigma-Aldrich) using the oxidative fluorescent dye dihydroethidine (DHE). The images were captured using a laser scanning confocal microscope system (Carl Zeiss LSM 900, Oberkochen, Germany).

### 2.5. Western Blot Analysis

Proteins from liver tissues and HepG2 cell lines were isolated using the Pro-Prep Protein Extraction Solution (iNtRON Biotechnology, Seongnam-si, Republic of Korea), adhering to the protocol provided by the supplier. These proteins were then subjected to separation via sodium dodecyl sulfate–polyacrylamide gel electrophoresis (SDS-PAGE) and subsequently transferred onto nitrocellulose membranes. The detection process involved the use of primary antibodies, including SOD1 (Enzo Life Sciences, Farmingdale, NY, USA), SOD2 (Enzo Life Sciences), SOD3 (Santa Cruz Biotechnology, Dallas, TX, USA), total AMPK (Cell Signaling Technology, Danvers, MA, USA), phospho-AMPK (Cell Signaling Technology), total FoxO1 (Cell Signaling Technology), phospho-FoxO1 (Cell Signaling Technology), PGC-1α (Novus Biologicals, Centennial, CO, USA), perilipin-2 (Abcam), peroxisome proliferator-activated receptorα (PPARα) (Abcam), PPARγ (Abcam), total acetyl-CoA carboxylase (ACC) (Santa Cruz Biotechnology), phospho-ACC (Santa Cruz Biotechnology), sterol regulatory element binding protein-1c (SREBP-1c) (Santa Cruz Biotechnology), carbohydrate response element binding protein (ChREBP) (Novus Biologicals), B cell leukemia/lymphoma 2 (Bcl-2) (Santa Cruz Biotechnology), Bcl-2-associated X (BAX) (Santa Cruz Biotechnology), beclin-1 (Novus Biolobicals), light chain3 (LC3) II (Sigma), monocyte chemoattractant protein-1 (MCP-1) (Abcam), TNF-α (Abcam), IL-6 (Proteintech, Rosemont, IL, USA), CD68 (Bio-Rad Laboratories, Hercules, CA, USA), granulocyte differentiation antigen-1 (Gr-1) (Bio-Rad Laboratories), arginase I (Santa Cruz Biotechnology), arginase II (Santa Cruz Biotechnology), inducible nitric oxide synthase (iNOS) (Novus Biologicals), Calcium/Calmodulin-Dependent Protein Kinase Kinase (CaMKK)α/β (Santa Cruz Biotechnology), total liver kinase B1 (LKB1) (Cell Signaling Technology), phospho-Ser^428^ LKB1 (Cell Signaling Technology) and GAPDH (Santa Cruz Biotechnology). Following the washing step, the membrane underwent incubation with either anti-mouse IgG or anti-rabbit IgG HRP-conjugated secondary antibodies provided by Cell Signaling Technology. The detection of the target proteins was achieved using an enhanced chemiluminescence substrate (ECL Plus; GE Healthcare Bio-Science, Piscataway, NJ, USA) and visualized with a Vilber chemiluminescence analyzer (Fusion SL 4; Vilber Lourmat, Marne-la-Vallée, France). Band densities were quantitatively analyzed using Quantity One software 4.5 (Bio-Rad Laboratory, Hercules, CA, USA).

### 2.6. In Vitro Study

The HepG2 human hepatocellular carcinoma cell line was acquired from the American Type Culture Collection (ATCC, Rockville, MD, USA) and was propagated following the ATCC’s guidelines. The growth medium for HepG2 cells consisted of Eagle’s Modified Minimum Essential Media (EMEM) enhanced with 10% fetal bovine serum (FBS) and 1% penicillin–streptomycin, both sourced from the ATCC. The cells were incubated at a constant temperature of 37 °C with 5% CO_2_/95% air and maintained at over 85% relative humidity. Cultivation of the cells in T-75 flasks was conducted weekly when they reached approximately 80% confluence, and the medium was replaced an additional time each week. Cells at passages 4–8 were used for all the experiments. HepG2 cells were exposed to low-glucose (LG) (5 mmol/L D-glucose), high-glucose (HG) (35 mmol/L D-glucose), palmitic acid (PA) (500 μM, Sigma-Aldrich) and HG+PA, with or without the additional 70 h administration of hEC-SOD (0.1 or 0.5 U/mL). Knockdown of *AMPKα1* (#5562-1), *AMPKα2* (#5563-2) and the negative control (#SN-1003) was performed using predesigned small interfering RNA (siRNA) sequences purchased from Bioneer (Daejeon, Republic of Korea). Transfection of *siAMPKα1* and *α2* was performed using Lipofectamine RNAiMax (Thermo Scientific), according to the manufacturer’s instructions. After transfection, cells were treated with hEC-SOD (0.5 U/mL) for 24 h.

### 2.7. Statistical Analysis

The results are presented as the mean and standard deviation. For multiple comparisons, ANOVA along with Bonferroni adjustment was utilized, employing SPSS 21.0 software (IBM, Armonk, NY, USA). A *p* value of less than 0.05 was deemed to indicate a statistically significant difference.

## 3. Results

### 3.1. Characteristics of Experimental Mice Groups

The body weight of *db*/*db* mice was significantly heavier than that of *db*/*m* and *db*/*m* hEC-SOD mice (*p* < 0.001), while a decrease in body weight was observed in *db*/*db* hEC-SOD mice (*p* < 0.001). The liver weight was markedly increased in *db*/*db* mice (*p* < 0.001), after having been reduced with hEC-SOD treatment (*p* = 0.001). Epididymal fat mass also exhibited a significant difference following hEC-SOD administration (*p* < 0.001). The serum level of fasting blood glucose and HbA1c were significantly higher in *db*/*db* and *db*/*db* hEC-SOD mice compared with *db*/*m* and *db*/*m* hEC-SOD mice (*p* < 0.001). While hEC-SOD treatment did not have an impact on blood glucose levels in diabetic mice, it notably reduced serum insulin levels and improved HOMA_IR_ in *db*/*db* mice (*p* < 0.001). Urinary isoprostane and 8-OH-dG and serum MCP-1 and tumor necrosis factor-α (TNF-α) levels decreased significantly following hEC-SOD treatment in *db*/*db* mice (*p* < 0.001). hEC-SOD administration led to a significant reduction in the serum levels of alanine transaminase and aspartate transaminase in *db*/*db* mice (*p* < 0.001) (Table 1).

### 3.2. Effects of hEC-SOD on Intrahepatic Histologic Changes Associated with Fibrosis, Inflammation and Lipid Accumulation

**Figure 1:**
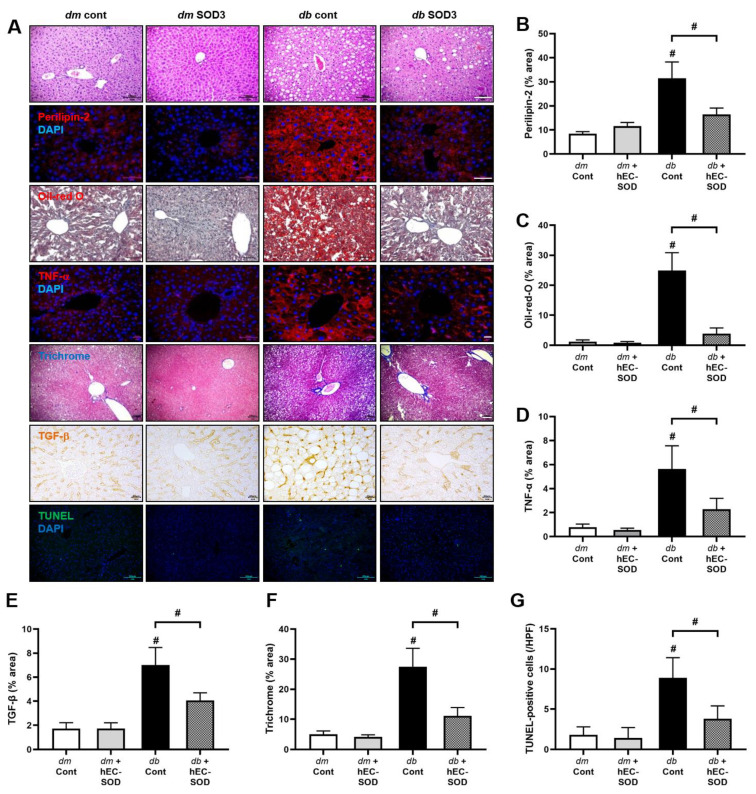
Effects of hEC-SOD treatment on liver phenotypes in *db*/*m* and *db*/*db* mice. The figure shows the liver steatosis, inflammation and fibrosis markers in different groups of mice. (**A**) Representative sections of hematoxylin and eosin stain, Oil Red O stain, Masson’s trichrome staining, immunofluorescence staining for perilipin-2, TNF-α and TUNEL and immunohistochemical staining for TGF-β1 are displayed in representative sections. The quantification of the results is presented for (**B**) Perilipin-2, (**C**) Oil Red O stain, (**D**) TNF-α, (**E**) TGF-β1, (**F**) trichrome staining and (**G**) TUNEL-positive cells. ^#^ *p* < 0.001 vs. *db*/*m* (*db*/*m* Cont), *dm* cont (*db*/*m* Cont), *dm* + hEC-SOD (*db*/*m* hEC-SOD) and *db* + hEC-SOD (*db*/*db* hEC-SOD) mice. hEC-SOD, human recombinant extracellular superoxide dismutase; TGF-β, transforming growth factor-β; TNF-α, tumor necrosis factor-α; TUNEL, terminal deoxynucleotidyl transferase-mediated dUTP nick end-labeling.

Figure 1 provides compelling evidence of a notable improvement in liver histology resulting from hEC-SOD treatment in *db*/*db* mice. The degree of hepatic steatosis, characterized by macrovesicular steatosis with prominent fat droplets, did not differ significantly between *db*/*m* and *db*/*m* hEC-SOD mice and was further increased in *db*/*db* mice. Notably, hEC-SOD treatment in *db*/*db* mice led to a marked reduction in hepatic steatosis (Figure 1A). This was corroborated by a substantial decrease in the accumulation of intrahepatic lipid droplets, as evidenced by the reduced levels of perilipin-2 and Oil Red O staining in *db*/*db* mice following hEC-SOD treatment (Figure 1A–C, *p* < 0.001 and *p* < 0.001, respectively). Furthermore, hEC-SOD treatment resulted in significant improvements in hepatic inflammation and fibrosis, as evidenced by TNF-α, trichrome and TGF-β staining (Figure 1A,D–F, *p* < 0.001, *p* < 0.001 and *p* < 0.001, respectively). In line with this, TUNEL-positive cells in the hepatocytes of all experimental groups were counted and analyzed. The number of TUNEL-positive hepatocytes in *db*/*db* mice was significantly attenuated by hEC-SOD treatment (Figure 1A,G, *p* < 0.001).

### 3.3. Intrahepatic SOD Isoforms and SOD3 Expression in Response to hEC-SOD Treatment

**Figure 2:**
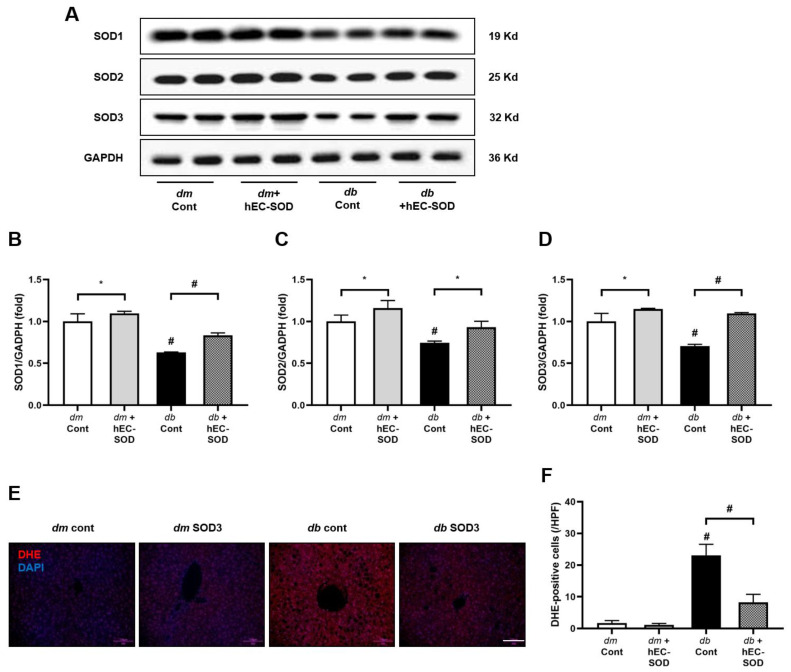
Analysis of hepatic SOD isoforms and oxidative stress in *db*/*m* and *db*/*db* mice subjected to hEC-SOD treatment. (**A**) The expression levels of SOD1, SOD2, SOD3 and GAPDH are shown in representative Western blots. The relative protein levels of (**B**) SOD1/GAPDH, (**C**) SOD2/GAPDH and (**D**) SOD3/GAPDH were quantified (n = 2). (**E**) Representative images of DHE staining in liver sections. (**F**) The quantification of DHE fluorescence intensity. * *p* < 0.05 and ^#^ *p* < 0.001 vs. *db*/*m* (*db*/*m* Cont), *dm* cont (*db*/*m* Cont), *dm* + hEC-SOD (*db*/*m* hEC-SOD) and *db* + hEC-SOD (*db*/*db* hEC-SOD) mice. DHE; dihydroethidium; hEC-SOD, human recombinant extracellular superoxide dismutase.

The levels of intrahepatic SOD isoforms (SOD1, SOD2 and SOD3) were measured, as presented in Figure 2. Compared with *db*/*m* and *db*/*m* hEC-SOD mice, liver tissue from *db*/*db* mice exhibited significant reductions in the expression of SOD1, SOD2 and SOD3 (Figure 2A). Following hEC-SOD administration, there was a notable increase in the levels of all SOD isoforms (SOD-1, 2 and 3) in *db*/*db* mice, though this was lower than that in *db*/*m* and *db*/*m* hEC-SOD mice. (Figure 2A–D, *p* < 0.001, *p* = 0.015 and *p* < 0.001, respectively). These findings suggest that hEC-SOD treatment can recover the expression of SOD in the steatotic liver of diabetic mice. In addition to changes in intrahepatic SOD expression, DHE staining was conducted to assess the extent of ROS formation. DHE staining revealed an increase in ROS levels in *db*/*db* mice, and administration of hEC-SOD resulted in a significant reduction in ROS (Figure 2E,F, *p* < 0.001).

### 3.4. Effects of hEC-SOD on Intrahepatic Expression of Phospho/Total AMPK and Associated Downstream Signaling Pathways including Phospho-/Total FoxOs and PGC-1α

**Figure 3:**
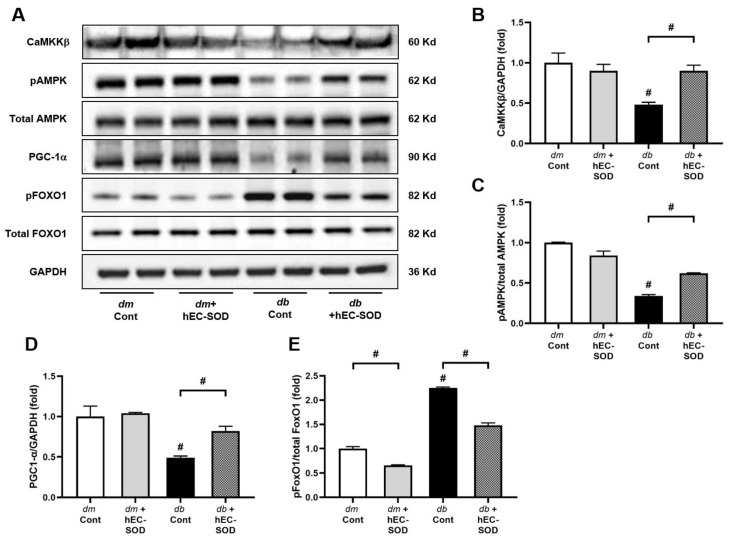
Expression of CaMKKβ, phospho-Thr^172^AMPK, total AMPK, PGC-1α, phospho-Ser^256^FoxO1 and total FoxO1 in *db*/*m* and *db*/*db* mice after hEC-SOD treatment. (**A**) The expression levels of CaMKKβ, phospho-Thr^172^AMPK, total AMPK, PGC-1α, phospho-Ser^256^FoxO1 and total FoxO1 are shown in representative Western blots. The relative protein levels of (**B**) CaMKKβ/GAPDH, (**C**) phospho-Thr^172^ AMPK/total AMPK, (**D**) PGC-1a/GAPDH and (**E**) phospho-Ser^256^ FoxO1/total FoxO1 were measured via densitometry (n = 2). ^#^ *p* < 0.001 vs. *db*/*m* (*db*/*m* Cont), *dm* cont (*db*/*m* Cont), *dm* + hEC-SOD (*db*/*m* hEC-SOD) and *db* + hEC-SOD (*db*/*db* hEC-SOD) mice. AMPK, adenosine monophosphate-activated protein kinase; CaMKKβ, Calcium/Calmodulin-Dependent Protein Kinase Kinase 2; FoxO, forkhead box O transcription factor; PGC-1α, peroxisome proliferative-activated receptor c coactivator 1α.

Insulin resistance and metabolic dysfunction markedly reduced phospho-Thr^172^ AMPK/total AMPK expression in the liver of *db*/*db* mice compared with that in the liver of *db*/*m* and *db*/*m* hEC-SOD mice. (Figure 3A). To further elucidate the action of SOD on the AMPK pathway, the expression of CaMKKβ, an upstream kinase of AMPK, was measured. In *db*/*db* mice, a significant decrease in the hepatic expression of CaMKKβ was observed, while there was a notable increase in the expression of this kinase in *db*/*db* hEC-SOD mice (Figure 3A,B, *p* < 0.001). Accordingly, treatment with hEC-SOD restored the phosphorylation of Thr^172^ AMPK/total AMPK levels to the levels of *db*/*m* and *db*/*m* hEC-SOD mice, indicating an increase in AMPK activity in *db*/*db* mice (Figure 3A,C, *p* < 0.001). The expression of PGC-1α and FoxO1, which are downstream targets of AMPK, was analyzed to investigate the changes in AMPK signaling. Decreased expression of PGC-1α was seen with hEC-SOD treatment in the liver of *db*/*db* mice groups (Figure 3A,D, *p* < 0.001). Increased expression of pFoxO1 was attenuated by hEC-SOD treatment in the liver of *db*/*db* mice groups (Figure 3A,E, *p* < 0.001). These findings suggest that hEC-SOD treatment activated AMPK and brought about changes in the expression of associated downstream signaling targets including FoxO and PGC-1α.

### 3.5. Effects of EC-SOD on Intrahepatic Expression of Perilipin-2, PPARα/γ, Phospho-ACC and SREBP-1c

**Figure 4:**
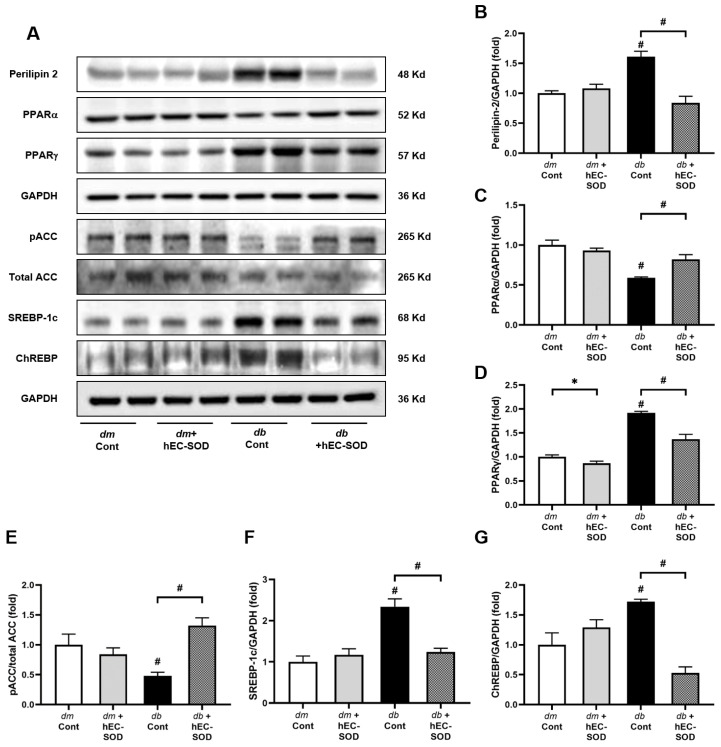
Expression of perilipin-2, PPARα, PPARγ, phospho-ACC, SREBP-1c and ChREBP in *db*/*m* and *db*/*db* mice after hEC-SOD treatment. (**A**) The expression levels of perilipin-2, PPARα, PPARγ, phospho-ACC, SREBP-1c and ChREBP are shown in representative Western blots. The relative protein levels of (**B**) perilipin-2/GAPDH, (**C**) PPARα/GAPDH, (**D**) PPARγ/GAPDH, (**E**) phospho-ACC/total ACC, (**F**) SREBP-1c/GAPDH and (**G**) ChREBP/GAPDH were measured via densitometry (n = 2). * *p* < 0.05 and ^#^ *p* < 0.001 vs. *db*/*m* (*db*/*m* Cont), *dm* cont (*db*/*m* Cont), *dm* + hEC-SOD (*db*/*m* hEC-SOD) and *db* + hEC-SOD (*db*/*db* hEC-SOD) mice. ACC, acetyl-CoA carboxylase; ChREBP, carbohydrate response element binding protein; PPAR, peroxisome proliferator-activated receptor; SREBP-1c, Sterol Regulatory Element Binding Protein-1.

The expression of perilipin-2 decreased following hEC-SOD treatment in the liver of *db*/*db* mice (Figure 4A,B, *p* < 0.001). Furthermore, hEC-SOD treatment improved the decreased expression of PPAR-α (Figure 4A,C, *p* < 0.001). Conversely, PPAR-γ expression increased in *db*/*db* mice and decreased after hEC-SOD treatment, indicating that hEC-SOD is involved in the PPAR pathway (Figure 4A,D, *p* < 0.001). Along with the upstream changes, which involved the activation of PGC-1α and PPARα, as well as the attenuation of PPARγ, phosphorylation of ACC was significantly increased (Figure 4A,E, *p* < 0.001). Additionally, the expression of SREBP-1c and ChREBP was significantly decreased in the liver of *db*/*db* mice following hEC-SOD treatment (Figure 4A,F,G, *p* < 0.001 and *p* < 0.001, respectively). These findings suggest that the prometabolic effect is anticipated, with fatty acid oxidation and mitochondrial biogenesis increasing in the liver tissue.

### 3.6. Effect of EC-SOD on Intrahepatic Expression of Bcl-2, BAX, Beclin-1 and LC3

**Figure 5:**
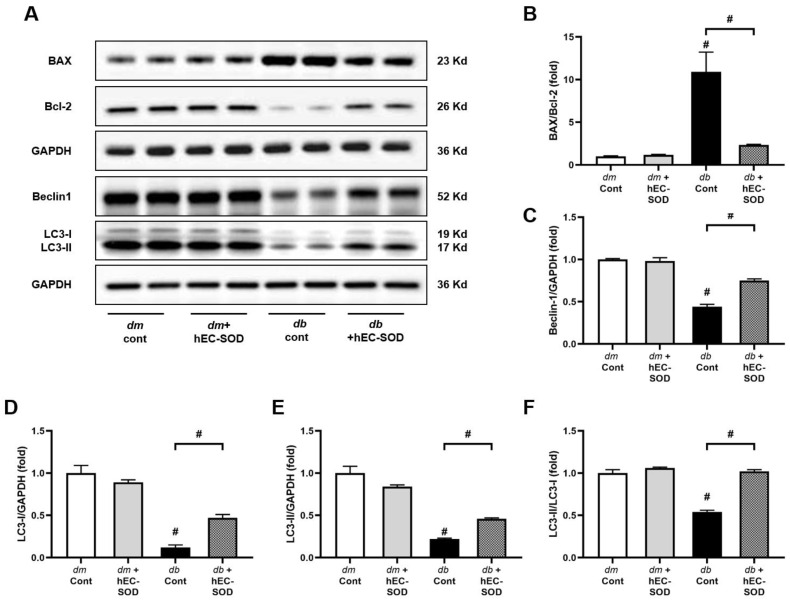
Effects of hEC-SOD treatment on apoptosis markers in *db*/*m* and *db*/*db* mice. (**A**) The expression levels of BAX, Bcl-2, Beclin-1 and LC3 are shown in representative Western blots. The relative protein levels of (**B**) BAX/Bcl-2, (**C**) Beclin-1/GAPDH, (**D**) LC3-I/GAPDH, (**E**) LC3-II/GAPDH and (**F**) LC3-II/LC3-1 were measured via densitometry (n = 2). ^#^ *p* < 0.001 vs. *db*/*m* (*db*/*m* Cont), *dm* cont (*db*/*m* Cont), *dm* + hEC-SOD (*db*/*m* hEC-SOD) and *db* + hEC-SOD (*db*/*db* hEC-SOD) mice. BAX, Bcl-2-associated X; Bcl-2, B cell leukemia/lymphoma 2; LC3, light chain3.

The antiapoptotic and pro-autophagy effects of hEC-SOD treatment were determined. The expression of Bcl-2 and BAX, which are involved in the regulation of apoptosis, was altered in the liver of *db*/*db* mice (Figure 5A). The antiapoptotic protein Bcl-2 was downregulated, while the proapoptotic protein BAX was upregulated. The increase in the BAX/Bcl-2 ratio was suppressed by the administration of hEC-SOD in the liver of *db*/*db* mice (Figure 5A,B, *p* < 0.001). Subsequently, the expression of autophagy-related proteins, Beclin-1 and LC3, which are regulated by AMPK activation, was determined. Suppressed expression of Beclin-1 and LC3 proteins was recovered by hEC-SOD treatment in the liver of *db*/*db* mice (Figure 5A,C–F, *p* < 0.001, *p* < 0.001, *p* < 0.001 and *p* < 0.001, respectively), suggesting a pro-autophagy effect of hEC-SOD.

### 3.7. Inflammatory Cytokines and Cellular Alterations in the Liver Following EC-SOD Treatment

**Figure 6:**
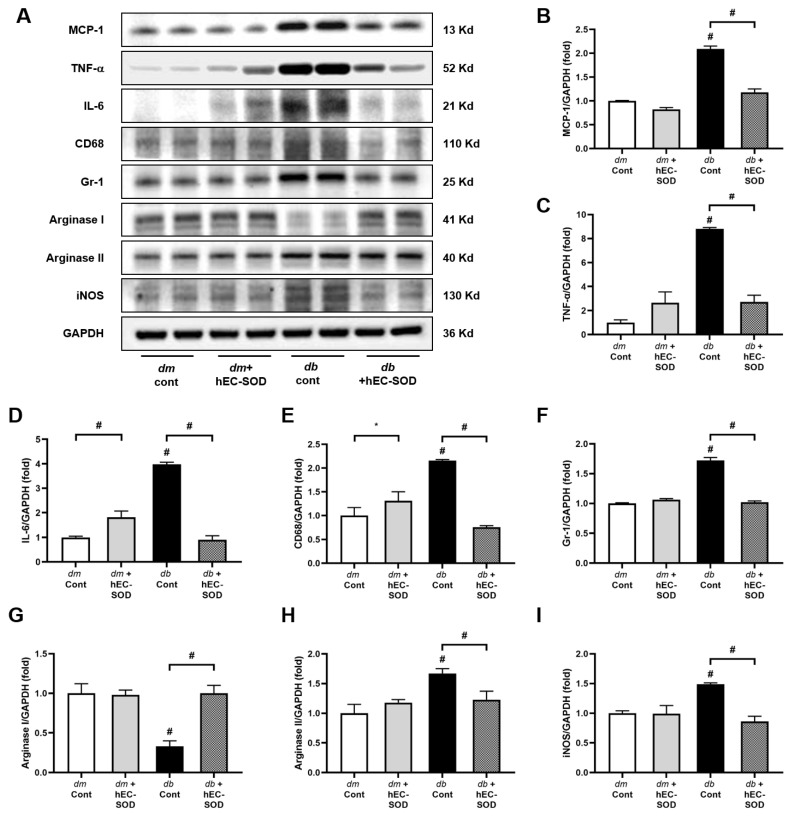
Effects of hEC-SOD treatment on inflammatory markers in *db*/*m* and *db*/*db* mice. (**A**) The expression levels of MCP-1, TNF-α, IL-6, CD68, Gr-1, Arginase I/II and iNOS are shown in representative Western blots. The relative protein levels of (**B**) MCP-1/GAPDH, (**C**) TNF-α/GAPDH, (**D**) IL-6/GAPDH, (**E**) CD68/GAPDH, (**F**) Gr-1/GAPDH, (**G**) Arginase I/GAPDH, (**H**) Arginase II/GAPDH and (**I**) iNOS/GAPDH were measured via densitometry (n = 2). * *p* < 0.05 and ^#^ *p* < 0.001 vs. *db*/*m* (*db*/*m* Cont), *dm* cont (*db*/*m* Cont), *dm* + hEC-SOD (*db*/*m* hEC-SOD) and *db* + hEC-SOD (*db*/*db* hEC-SOD) mice. Gr-1, granulocyte differentiation antigen-1; iNOS, inducible nitric oxide synthase; MCP-1, monocyte chemoattractant protein-1; TNF-α, tumor necrosis factor-α.

Intrahepatic expression of MCP-1, TNF-α and IL-6 in the liver of *db*/*db* mice was restored by treatment with hEC-SOD to the levels of those in *db*/*m* and *db*/*m* hEC-SOD (Figure 6A–D, *p* < 0.001, *p* < 0.001 and *p* < 0.001, respectively). These findings suggest that hEC-SOD treatment ameliorated the inflammatory conditions in *db*/*db* mice. Increased expression of CD68 and Gr-1 in the liver of *db*/*db* mice was reduced by hEC-SOD treatment (Figure 6A,E,F, *p* < 0.001 and *p* < 0.001, respectively). In *db*/*db* mice, the expression of arginase I was significantly decreased, while the expression of arginase II increased. However, upon treatment with EC-SOD, these changes were reversed, with arginase II expression markedly decreasing and arginase I expression increasing (Figure 6A,G,H, *p* < 0.001 and *p* < 0.001, respectively). The level of iNOS, which is an indicator of macrophage M1 polarization, was elevated in *db*/*db* mice and significantly reduced by hEC-SOD treatment (Figure 6A,I, *p* < 0.001). These results indicate that EC-SOD attenuates hepatic inflammation in a steatotic liver by decreasing the production of proinflammatory cytokines from macrophage M1 polarization, without affecting macrophage M2 polarization.

### 3.8. In Vitro Studies (HepG2)

**Figure 7:**
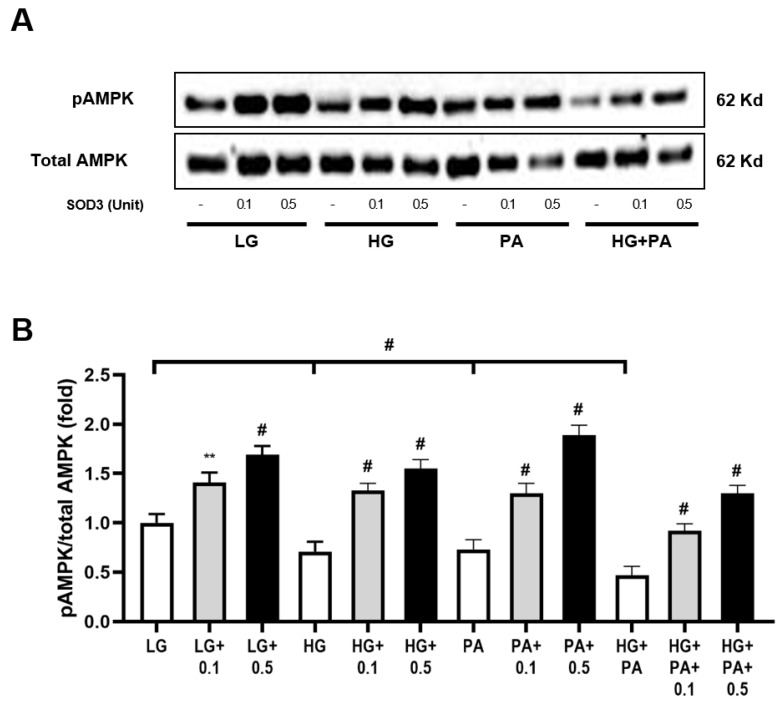
Dose-dependent effects of hEC-SOD treatment on AMPK expression in HepG2 cells exposed to different media. HepG2 cells were cultured with LG, HG or PA media and treated with various doses of hEC-SOD. (**A**) The expression levels of phospho-Thr^172^AMPK and total AMPK are shown in representative Western blots. (**B**) The relative protein levels of phospho-Thr^172^ AMPK/total AMPK were measured via densitometry (n = 3). ** *p* < 0.01 and ^#^ *p* < 0.001 compared with other groups. AMPK, adenosine monophosphate-activated protein kinase; HG, high-glucose; LG, low-glucose; PA, palmitic acid.

**Figure 8:**
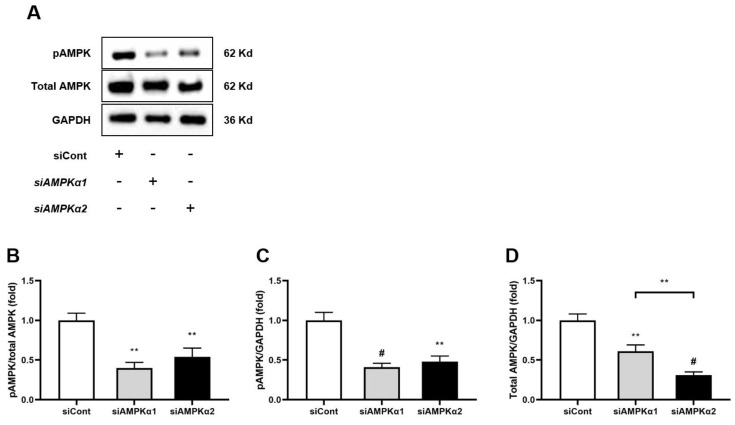
Effects of siRNA for *AMPKα1* or *AMPKα2* on AMPK expression in HepG2 cells. HepG2 cells were treated with transfection reagent and 50 nM of control siRNA, *AMPKα1* siRNA or *AMPKα2* siRNA. (**A**) The expression levels of phospho-Thr^172^AMPK and total AMPK are shown in representative Western blots. The relative protein levels of (**B**) phospho-Thr^172^ AMPK/total AMPK, (**C**) phospho-Thr^172^ AMPK/GAPDH and (**D**) total AMPK/GAPDH were measured via densitometry (n = 3). ** *p* < 0.01 and ^#^ *p* < 0.001 compared with other groups.

The effect of hEC-SOD treatment on HepG2 cells, a human hepatoma cell line, was evaluated in terms of inflammation, oxidative stress and apoptosis. A significant decrease in the ratio of phosphorylated-Thr^172^ AMPK to total AMPK was observed in the HG, PA and HG+PA groups compared with the LG-treated group (Figure 7A,B, *p* < 0.001, *p* < 0.001 and *p* < 0.001, respectively). Following the administration of hEC-SOD, the ratios of phosphorylated-Thr^172^ AMPK to total AMPK were significantly improved across all conditions. Moreover, these improvements exhibited a dose-dependent trend, with higher doses of hEC-SOD (0.1 to 0.5 U/mL) leading to greater increases in the ratios. (Figure 7A,B). This dose-dependent effect further substantiates that these observed changes are indeed a response to SOD. To further explore the role of hEC-SOD treatment in activating the AMPK pathway, experiments using siRNAs for *AMPKα1* and *AMPKα2* were conducted. The expression of both phospho-Thr^172^ AMPK and total AMPK was successfully suppressed in HepG2 cells that had been transfected with siRNAs for *AMPKα1* and *AMPKα2* (Figure 8A–D, *p* < 0.01, *p* < 0.01 and *p* < 0.01, respectively).

**Figure 9:**
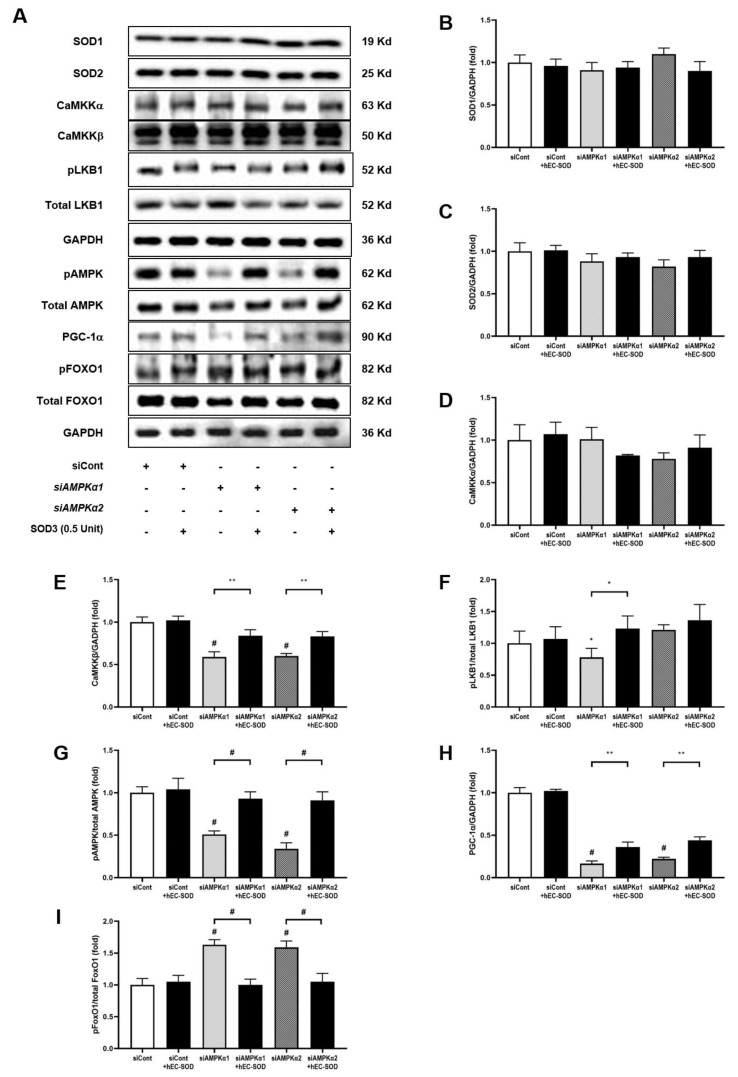
Effects of siRNA for *AMPKα1* or *AMPKα2* and hEC-SOD on SOD isoforms, CaMKKα/β, phospho-LKB1, total LKB1, phospho-Thr^172^AMPK, total AMPK, PGC-1α, phospho-Ser^256^FoxO1 and total FoxO1 in HepG2 cells. HepG2 cells were treated with transfection reagent and 50 nM of control siRNA, *AMPKα1* siRNA or *AMPKα2* siRNA. Then, the cells were exposed to 0.5 U/mL hEC-SOD. (**A**) Representative Western blot for SOD1, SOD2, CaMKKα, CaMKKβ, phospho-LKB1, total LKB1, phospho-Thr^172^AMPK, total AMPK, PGC-1a, phospho-Ser^256^FoxO1 and total FoxO1. The relative protein levels of (**B**) SOD1/GAPDH, (**C**) SOD2/GAPDH, (**D**) CaMKKα/GAPDH, (**E**) CaMKKβ/GAPDH, (**F**) phospho-LKB1/total LKB1, (**G**) phospho-Thr^172^AMPK/total AMPK, (**H**) PGC-1α/GAPDH and (**I**) phospho-Ser^256^FoxO1/total FoxO1 were quantified via densitometry (n = 3). * *p* < 0.05, ** *p* < 0.01 and ^#^ *p* < 0.001 compared with other groups. CaMKK, calcium/calmodulin-dependent protein kinase kinase; LKB1, liver kinase B1.

There were no significant differences observed in the expression levels of SOD1 and SOD2 in HepG2 cells after treatment with siRNAs for *AMPKα1* and *AMPKα2* (Figure 9A–C). While the expression of CaMKKα did not exhibit significant differences (Figure 9A,D), the expression of CaMKKβ was significantly decreased in in the groups treated with siRNAs for *AMPKα1* and *AMPKα2* (Figure 9A,E, *p* < 0.001 and *p* < 0.001, respectively). This suggests that the knockdown of *AMPKα1* and *AMPKα2* was specifically associated with CaMKKβ. Notably, the reduced expression of CaMKKβ was significantly ameliorated following the administration of hEC-SOD (Figure 9A,E, *p* < 0.01 and *p* < 0.01, respectively). The ratio of phosphorylated liver kinase B1 (LKB1) to total LKB1 decreased upon treatment with siRNAs for *AMPKα1*, but this was reversed after the administration of hEC-SOD (Figure 9A,F, *p* < 0.05 and *p* < 0.05, respectively). Consistent with the observed changes in CaMKKβ expression, transfection with siRNAs for *AMPKα1* and *AMPKα2* in HepG2 cells resulted in a significant decrease in the ratio of phospho-Thr^172^ AMPK to total AMPK and the expression PGC-1α (Figure 9A,G,H, *p* < 0.001 and *p* < 0.001, respectively). The decreases in the pAMPK/total AMPK ratio and PGC-1α expression were significantly improved in the hEC-SOD-treated group (Figure 9A,G,H, *p* < 0.001 and *p* < 0.01, respectively). Phosphorylation of FoxO1 was significantly increased by siRNAs for *AMPKα1* and *AMPKα2* but ameliorated by hEC-SOD treatment (Figure 9A,I, *p* < 0.001 and *p* < 0.01, respectively).

**Figure 10:**
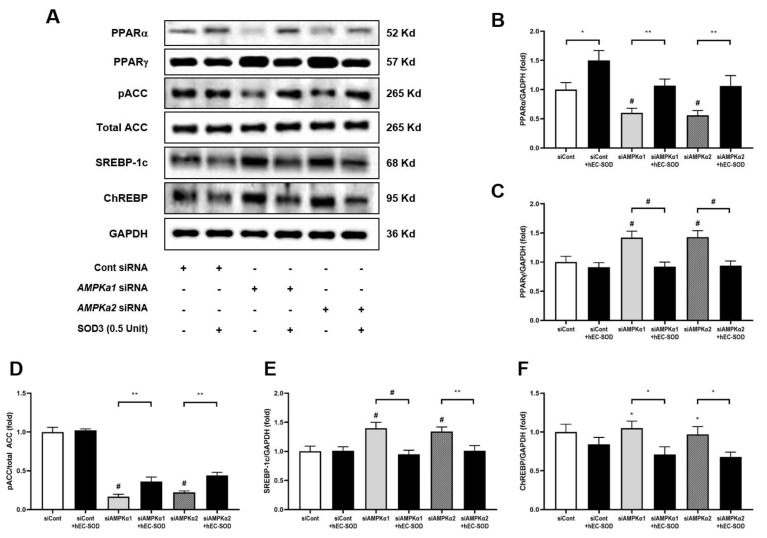
Effects of siRNA for *AMPKα1* or *AMPKα2* and hEC-SOD on PPARα/γ, phospho-ACC, total ACC, SREBP-1c and ChREBP in HepG2 cells. HepG2 cells were treated with transfection reagent and 50 nM of control siRNA, *AMPKα1* siRNA or *AMPKα2* siRNA. Then, the cells were exposed to 0.5 U/mL hEC-SOD. (**A**) Representative Western blot for PPARα/γ, phospho-ACC, total ACC, SREBP-1c and ChREBP. The relative protein levels of (**B**) PPARα/GAPDH, (**C**) PPARγ/GAPDH, (**D**) phospho-ACC/total ACC, (**E**) SREBP-1c/GAPDH and (**F**) ChREBP-1c/GAPDH were quantified via densitometry (n = 3). * *p* < 0.05, ** *p* < 0.01 and ^#^ *p* < 0.001 compared with other groups.

Transfection with siRNAs for *AMPKα1* and *AMPKα2* resulted in the suppression of PPAR-α expression while enhancing PPAR-γ expression. (Figure 10A–C, *p* < 0.001 and *p* < 0.001, respectively). These observations were reversed upon administration of hEC-SOD, leading to an increase in PPAR-α and a decrease in PPAR-γ expression (Figure 10A–C, *p* < 0.01 and *p* < 0.001, respectively). In the context of the PPAR family’s downstream pathway, further examinations were conducted for phospho-ACC, SREBP-1c and ChREBP. The transfection of siRNAs for *AMPKα1* and *AMPKα2* led to a marked reduction in phospho-ACC levels but the upregulation of SREBP-1c and ChREBP expression (Figure 10A,D–F, *p* < 0.001, *p* < 0.001 and *p* < 0.05, respectively). Administration of hEC-SOD reversed these effects, resulting in elevated phospho-ACC levels and a reduction in the expression of both SREBP-1c and ChREBP (Figure 10A,D–F, *p* < 0.01, *p* < 0.01 and *p* < 0.05, respectively).

**Figure 11:**
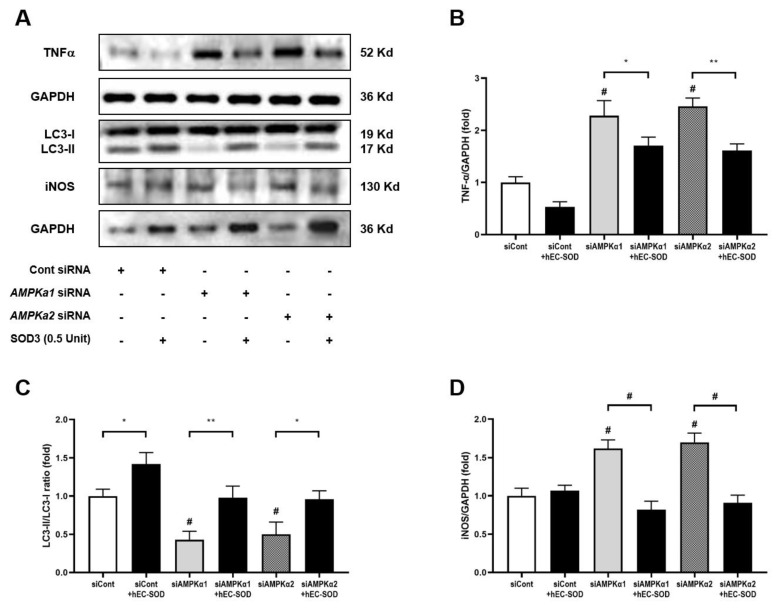
Effects of siRNA for *AMPKα1* or *AMPKα2* and hEC-SOD on TNF-α, LC-3 and iNOS in HepG2 cells. HepG2 cells were treated with transfection reagent and 50 nM of control siRNA, *AMPKα1* siRNA or *AMPKα2* siRNA. Then, the cells were exposed to 0.5 U/mL hEC-SOD. (**A**) Representative Western blot for TNF-α, LC-3 and iNOS. The relative protein levels of (**B**) TNF-α/GAPDH, (**C**) LC3-I/LC3-II and (**D**) iNOS/GAPDH were quantified via densitometry (n = 3). * *p* < 0.05, ** *p* < 0.01 and ^#^ *p* < 0.001 compared with other groups.

In HepG2 cells transfected with siRNA for *AMPKα1* and *AMPKα2*, hEC-SOD treatment significantly attenuated the increased expression of TNF-α (Figure 11A,B, *p* < 0.05). This treatment also restored the suppressed LC3-II/LC3-I ratio (Figure 11A,C, *p* < 0.05) and reduced the elevated levels of iNOS (Figure 11A,D, *p* < 0.001). These findings suggest a potential regulatory role of hEC-SOD in modulating inflammation and autophagy in the context of *AMPKα1* and *AMPKα2* knockdown.

## 4. Discussion

Our studies showed that EC-SOD might be a potential therapeutic agent for NAFLD through the direct activation of AMPK-PGC-1α and AMPK-FoxO1 signaling in hepatocytes, which modulates lipid metabolism, leading to anti-inflammatory, antioxidative and antiapoptotic effects and improving autophagy in the liver. This study holds significance for unveiling the antioxidant effects and metabolic insights associated with EC-SOD in NAFLD, establishing it as a potential therapeutic target. In a study by Cui et al., the overexpression of SOD3 in C57BL/6 mice subjected to a high-fat diet (HFD) effectively blocked obesity, fatty liver and insulin resistance [11]. Our study provides compelling evidence of the amelioration of steatosis with hEC-SOD administration, demonstrating histological improvements that extend to steatosis and fibrosis through activating AMPK and its associated downstream targets involved in lipid metabolism (Figure 1). To the best of our knowledge, this is the first report on the therapeutic impact of hEC-SOD in ameliorating hepatic steatosis.

SOD3, a member of the SOD family, has potential as a biopharmaceutical agent for inflammatory diseases [21]. It can attenuate inflammation by reducing ROS levels and modulating cellular signals. Moreover, SOD3 does not need intracellular translocation, unlike SOD1 and SOD2. Furthermore, SOD3 has a longer circulation half-life of about 20 h compared with SOD1 and SOD2, which have half-lives of about 20 min and 5–6 h, respectively [22,23]. SOD3 has demonstrated its protective role in various tissues, including the lung, kidney, skin and retina, by mitigating the effects of oxidative stress [20,24,25,26]. Gao et al. provided evidence that SOD3 serves as a protective factor secreted by adipocytes in response to HFD-induced obesity [10]. Their study revealed a significant upregulation of SOD3 expression in the adipose tissue of HFD-fed mice. Notably, Sod3 knockout mice exhibited an altered phenotype characterized by increased obesity, insulin resistance, enlarged adipose tissue and elevated triglyceride accumulation. This study revealed a significant reduction in hepatic EC-SOD expression in a steatotic liver, which was ameliorated through the administration of hEC-SOD, as confirmed with Western blot and immunohistochemical staining (Figure 2A–D). Our results show improvements in oxidative stress markers, which include 24 h urinary isoprostane, a recognized measure of oxidative stress, and 24 h urinary 8-hydroxy-deoxyguanosine, a marker indicative of DNA damage resulting from oxidative stress (Table 1). Furthermore, quantitative assessment of ROS levels in liver tissue using DHE staining demonstrated a significant reduction in ROS in response to hEC-SOD administration (Figure 2E,F). These improvements extended to a reduction in systemic and hepatic inflammatory markers, including MCP-1 and TNF-α, following hEC-SOD administration (Figure 6).

NAFLD exhibits a multitude of clinical phenotypes and considerable heterogeneity owing to the intricate nature of its pathogenesis and diverse clinical circumstances [27]. This condition has the potential to advance to more severe complications, including liver cirrhosis and hepatocellular carcinoma, driven by inflammation and fibrosis. Although effective treatments have been established for other components of the metabolic syndrome, including diabetes, hypertension, hyperlipidemia and even obesity, there is a notable absence of specific pharmaceutical interventions for NAFLD at present [28]. In a landscape where current treatments have limited effectiveness, antioxidants are gaining attention as a potential new therapeutic target [29,30]. The application of SOD, which has been investigated in various animal models and clinical trials, holds promise across a spectrum of conditions, from hypoxic damage and cardiovascular diseases to neurodegenerative disorders and metabolic diseases [31]. 

Sun et al. showed that EC-SOD was involved in liver damage through the AMPK pathway, even without metabolic dysfunction [32]. They reported that Sod3^−/−^ mice exhibited spontaneous liver injury and fibrosis. Notably, their findings suggest that SOD3 deficiency exacerbated liver fibrosis by suppressing AMPK signaling. Furthermore, in a previous study by our research team, the administration of hEC-SOD was shown to improve diabetic nephropathy through AMPK-PGC-1α-Nrf2 and AMPK-FoxOs signaling pathways [20]. AMPK is a critical protein involved in regulating cellular energy metabolism and becomes active when cellular energy levels decrease. In a recent study, Wang et al. demonstrated that within the NAFLD model, CAMKKβ functions as the initiating kinase for the AMPK-mediated antioxidant defense mechanism, which protect hepatocytes from lipotoxicity [33]. In the current study, we observed a decrease in the expression of CAMKKβ in *db*/*db* mice, which was restored following hEC-SOD administration (Figure 3A,B). This finding provides significant validation of previous research results. PGC-1α is a transcriptional coactivator protein that plays a significant role in the mitochondrial life cycle and response to ROS. Stimuli that activate AMPK have been reported to inhibit FoxO1-dependent transcription [34]. In the current study, we observed an improvement in the pAMPK/total AMPK ratio and the expression of PGC-1α in diabetic mice after hEC-SOD administration (Figure 3). Additionally, hEC-SOD treatment significantly reduced pFoxO1 expression in *db*/*db* mice. These findings suggest that targeting the AMPK-FoxOs signaling pathway through EC-SOD activation could be advantageous in mitigating intracellular oxidative stress and apoptosis in a steatotic liver. In the same line, our data strongly indicate that EC-SOD directly contributes to the reduction in oxidative stress by modulating the AMPK pathway.

PPARs are nuclear receptors involved in regulating lipid metabolism and inflammation. Among the various PPAR subtypes, PPARγ plays a pivotal role in promoting hepatic steatosis by increasing the expression of SREBP-1c [35]. This upregulation leads to heightened synthesis of fatty acids and cholesterol in the liver. Additionally, the downregulation of PPARγ results in the phosphorylation of ACC, a key enzyme in fatty acid synthesis, resulting in a decrease in lipogenesis [36]. In our study, diabetic mice exhibited an increase in the expression of PPARγ, along with reduced phosphorylation of ACC and elevated expression of SREBP-1c and ChREBP (Figure 4). Following hEC-SOD administration, we observed a decrease in PPARγ along with an attenuation of SREBP-1c and an increase in ACC phosphorylation. This evidence suggests the inhibition of fatty acid synthesis in the liver.

One of the key factors in the mechanism of liver damage due to oxidative stress is the increase in apoptosis. The Bax/Bcl-2 ratio is a crucial indicator of the balance between pro-apoptotic and antiapoptotic proteins in the Bcl-2 family [37]. We found a reduction in apoptosis as indicated by the decreased Bax/Bcl-2 ratio with SOD3 administration (Figure 5). This indicates that the administration of hEC-SOD improved the apoptotic environment. Furthermore, we observed a decrease in MCP-1, TNF-α, IL-6, CD68 and Gr-1 following hEC-SOD administration (Figure 6). These results indicate that hEC-SOD has both antioxidant and anti-inflammatory effects locally and systemically. Additionally, a decrease in iNOS and arginase II, along with an increase in arginase I, indicates macrophage polarization from M1 to M2, contributing to inflammation resolution and tissue repair in the liver.

A key discovery in our research was a detailed understanding of the molecular mechanism of SOD3’s function in hepatocytes, as determined through rigorous in vitro analyses. Upon the application of *AMPKα1* and *AMPKα2* siRNA to HepG2 cells, there was a significant reduction in CAMKKβ expression and AMPK phosphorylation. However, these results were restored with the administration of hEC-SOD (Figure 9). Moreover, the restorative effect of hEC-SOD on pAMPK was dose-dependent, with higher hEC-SOD doses leading to greater ratio increases (Figure 7). In alignment with our previously mentioned in vivo study results, we observed that various components of the AMPK downstream pathway, including PGC-1α, pFoxO1, PPAR α/γ, pACC, SREBP-1c and ChREBP, exhibited improvement subsequent to the administration of hEC-SOD.

The use of anti-hyperglycemic drugs such as pioglitazone, glucagon-like peptide 1 analogues and sodium-glucose cotransporter 2 inhibitors had raised expectations for their potential efficacy in improving NAFLD. However, it has been found that these drugs have limited effectiveness in achieving histologic improvements in NAFLD. However, our study demonstrated that the administration of hEC-SOD resulted in an improvement in hepatic steatosis independent of blood glucose levels. In our research, despite the absence of significant differences in blood glucose and HbA1C levels before and after hEC-SOD administration, an improvement in HOMA_IR_ was observed (Figure 1). EC-SOD was previously reported to protect against adipose tissue inflammation and insulin resistance [11]. Furthermore, serum EC-SOD levels were shown to be negatively correlated with insulin resistance in patients with type 2 diabetes [38,39,40]. In our study, administration of hEC-SOD not only reduced oxidative stress but also decreased insulin resistance. We hypothesize that these dual effects of EC-SOD contributed to the histological improvement in hepatic steatosis observed in our study. One of the major clinical unmet needs in NAFLD is the absence of biomarkers [41,42]. While there have been some past studies that measured SOD levels in the blood of patients with hepatic steatosis, these studies have not gained widespread recognition due to discrepancies in their results [43,44,45]. Several studies have been published on the utility of EC-SOD as a biomarker for hepatic failure in various liver diseases [46,47,48]. Considering the strong association observed between EC-SOD and hepatic steatosis in this study, it is conceivable that EC-SOD could be employed as a biomarker. Its use in conjunction with known biomarkers or measuring differences in its level before and after treatment provides a potential avenue for assessment.

## 5. Conclusions

Our study demonstrated the effectiveness of hEC-SOD treatment in improving steatotic liver disease. This improvement was closely associated with the activation of AMPK and its associated pathways, such as AMPK-PGC-1α and AMPK-FoxO1 phosphorylation, leading to improvements in lipid metabolism, oxidative stress and inflammation. These findings suggest that hEC-SOD might act as a potential therapeutic agent for lipotoxicity in NAFLD via the direct activation of AMPK in hepatocytes.

## Figures and Tables

**Table 1:** Biochemical and physical characteristics of the four groups at the end of the experimental period.

Variables	*db*/*m* Control	*db*/*m* hEC-SOD	*db*/*db* Control	*db*/*db* hEC-SOD
Body weight (g)	30.9 ± 1.6	30.2 ± 0.8	55.6 ± 2.7 ^†^	40.5 ± 4.8 ^‡^
Liver weight (g)	1.6 ± 0.3	1.4 ± 0.1	3.4 ± 0.9 ^†^	2.0 ± 0.1 **
Epididymal wt (g)	0.7 ± 0.2	0.4 ± 0.1	2.5 ± 0.3 ^†^	1.7 ± 0.2 ^‡^
Glucose (mg/dL)	149.7 ± 37.9	137.3 ± 18.4	543.8 ± 34.1 ^†^	537.3 ± 25.0 ^†^
HbA1c (%)	4.1 ± 0.1	3.9 ± 0.1	12.7 ± 1.1 ^†^	12.0 ± 0.6 ^†^
Insulin (ng/mL)	0.36 ± 0.06	0.37 ± 0.14	4.75 ± 1.32 ^†^	1.81 ± 0.69 ^‡^
HOMA_IR_	1.30 ± 0.40	1.42 ± 0.49	7.09 ± 2.31 ^†^	3.13 ± 0.86 ^‡^
Urinary 8-OH-dG (ng)	30.8 ± 1.6	26.8 ± 7.7	224.5 ± 73.1 ^†^	42.0 ± 16.6 ^‡^
Urinary isoprostane (ng/24 h)	4.2 ± 1.5	3.5 ± 0.6	44.5 ± 13.8 ^†^	18.5 ± 3.8 ^‡^
MCP-1 (pg/mL)	141.5 ± 11.9	146.5 ± 18.6	343.1 ± 38.0 ^†^	187.9 ± 28.7 ^‡^
TNF-α (pg/mL)	2.2 ± 0.3	2.1 ± 0.3	2.9 ± 0.4 ^†^	2.0 ± 0.2 ^‡^
ALT (U/L)	23.7 ± 6.8	21.7 ± 2.8	52.2 ± 8.5 ^†^	31.5 ± 5.9 ^‡^
AST (U/L)	27.5 ± 3.3	24.3 ± 6.9	49.2 ± 4.1 ^†^	32.2 ± 2.9 ^‡^

^†^ *p* < 0.001 compared with *db*/*m* control and *db*/*m* hEC-SOD groups and ** *p* < 0.05, ^‡^ *p* < 0.01 compared with *db*/*db* control. 8-OH-dG, 8-hydroxy-deoxyguanosine, hEC-SOD, human recombinant extracellular superoxide dismutase; HbA1C, glycosylated hemoglobin; HOMA_IR_, homeostatic model assessment for insulin resistance; MCP-1, monocyte chemoattractant protein-1; TNF-α, tumor necrosis factor-α; ALT, alanine transaminase; AST, aspartate aminotransferase.

## Data Availability

The original contributions presented in the study are included in the article; further inquiries can be directed to the corresponding author.

## References

[1] Tilg H., Moschen A.R., Roden M. (2017). NAFLD and diabetes mellitus. Nat. Rev. Gastroenterol. Hepatol..

[2] Peverill W., Powell L.W., Skoien R. (2014). Evolving concepts in the pathogenesis of NASH: Beyond steatosis and inflammation. Int. J. Mol. Sci..

[3] Masarone M., Rosato V., Dallio M., Gravina A.G., Aglitti A., Loguercio C., Federico A., Persico M. (2018). Role of Oxidative Stress in Pathophysiology of Nonalcoholic Fatty Liver Disease. Oxid. Med. Cell. Longev..

[4] Mitrofanova A., Merscher S., Fornoni A. (2023). Kidney lipid dysmetabolism and lipid droplet accumulation in chronic kidney disease. Nat. Rev. Nephrol..

[5] Lytrivi M., Castell A.L., Poitout V., Cnop M. (2020). Recent Insights Into Mechanisms of beta-Cell Lipo- and Glucolipotoxicity in Type 2 Diabetes. J. Mol. Biol..

[6] An Y., Xu B.T., Wan S.R., Ma X.M., Long Y., Xu Y., Jiang Z.Z. (2023). The role of oxidative stress in diabetes mellitus-induced vascular endothelial dysfunction. Cardiovasc. Diabetol..

[7] Fridovich I. (1997). Superoxide anion radical (O_2_^−^·), superoxide dismutases, and related matters. J. Biol. Chem..

[8] Yan Z., Spaulding H.R. (2020). Extracellular superoxide dismutase, a molecular transducer of health benefits of exercise. Redox Biol..

[9] Fattman C.L., Schaefer L.M., Oury T.D. (2003). Extracellular superoxide dismutase in biology and medicine. Free. Radic. Biol. Med..

[10] Gao D., Hu S., Zheng X., Lin W., Gao J., Chang K., Zhao D., Wang X., Zhou J., Lu S. (2020). SOD3 Is Secreted by Adipocytes and Mitigates High-Fat Diet-Induced Obesity, Inflammation, and Insulin Resistance. Antioxid. Redox Signal..

[11] Cui R., Gao M., Qu S., Liu D. (2014). Overexpression of superoxide dismutase 3 gene blocks high-fat diet-induced obesity, fatty liver and insulin resistance. Gene Ther..

[12] Kim Y., Park C.W. (2016). Adenosine monophosphate-activated protein kinase in diabetic nephropathy. Kidney Res. Clin. Pract..

[13] Fang C., Pan J., Qu N., Lei Y., Han J., Zhang J., Han D. (2022). The AMPK pathway in fatty liver disease. Front. Physiol..

[14] Wan Z., Root-McCaig J., Castellani L., Kemp B.E., Steinberg G.R., Wright D.C. (2014). Evidence for the role of AMPK in regulating PGC-1 alpha expression and mitochondrial proteins in mouse epididymal adipose tissue. Obesity.

[15] Zhang H., Zhu Y., Suehiro Y., Mitani S., Xue D. (2023). AMPK-FOXO-IP3R signaling pathway mediates neurological and developmental defects caused by mitochondrial DNA mutations. Proc. Natl. Acad. Sci. USA.

[16] Baldelli S., Aquilano K., Ciriolo M.R. (2014). PGC-1alpha buffers ROS-mediated removal of mitochondria during myogenesis. Cell Death Dis..

[17] Abu Shelbayeh O., Arroum T., Morris S., Busch K.B. (2023). PGC-1alpha Is a Master Regulator of Mitochondrial Lifecycle and ROS Stress Response. Antioxidants.

[18] Kim Y., Lim J.H., Kim M.Y., Kim E.N., Yoon H.E., Shin S.J., Choi B.S., Kim Y.S., Chang Y.S., Park C.W. (2018). The Adiponectin Receptor Agonist AdipoRon Ameliorates Diabetic Nephropathy in a Model of Type 2 Diabetes. J. Am. Soc. Nephrol..

[19] Hong Y.A., Lim J.H., Kim M.Y., Kim T.W., Kim Y., Yang K.S., Park H.S., Choi S.R., Chung S., Kim H.W. (2014). Fenofibrate improves renal lipotoxicity through activation of AMPK-PGC-1alpha in db/db mice. PLoS ONE.

[20] Hong Y.A., Lim J.H., Kim M.Y., Kim Y., Park H.S., Kim H.W., Choi B.S., Chang Y.S., Kim H.W., Kim T.Y. (2018). Extracellular Superoxide Dismutase Attenuates Renal Oxidative Stress Through the Activation of Adenosine Monophosphate-Activated Protein Kinase in Diabetic Nephropathy. Antioxid. Redox Signal..

[21] Kwon M.J., Han J., Kim B.H., Lee Y.S., Kim T.Y. (2012). Superoxide dismutase 3 suppresses hyaluronic acid fragments mediated skin inflammation by inhibition of toll-like receptor 4 signaling pathway: Superoxide dismutase 3 inhibits reactive oxygen species-induced trafficking of toll-like receptor 4 to lipid rafts. Antioxid. Redox Signal..

[22] Karlsson K., Sandstrom J., Edlund A., Edlund T., Marklund S.L. (1993). Pharmacokinetics of extracellular-superoxide dismutase in the vascular system. Free Radic. Biol. Med..

[23] Gorecki M., Beck Y., Hartman J.R., Fischer M., Weiss L., Tochner Z., Slavin S., Nimrod A. (1991). Recombinant human superoxide dismutases: Production and potential therapeutical uses. Free Radic. Res. Commun..

[24] Yao H., Arunachalam G., Hwang J.W., Chung S., Sundar I.K., Kinnula V.L., Crapo J.D., Rahman I. (2010). Extracellular superoxide dismutase protects against pulmonary emphysema by attenuating oxidative fragmentation of ECM. Proc. Natl. Acad. Sci. USA.

[25] Agrahari G., Sah S.K., Nguyen C.T., Choi S.S., Kim H.Y., Kim T.Y. (2020). Superoxide Dismutase 3 Inhibits LL-37/KLK-5-Mediated Skin Inflammation through Modulation of EGFR and Associated Inflammatory Cascades. J. Investig. Dermatol..

[26] Lee J.Y., Kim M., Oh S.B., Kim H.Y., Kim C., Kim T.Y., Park Y.H. (2022). Superoxide dismutase 3 prevents early stage diabetic retinopathy in streptozotocin-induced diabetic rat model. PLoS ONE.

[27] Takaki A., Kawai D., Yamamoto K. (2013). Multiple hits, including oxidative stress, as pathogenesis and treatment target in non-alcoholic steatohepatitis (NASH). Int. J. Mol. Sci..

[28] Rinella M.E., Neuschwander-Tetri B.A., Siddiqui M.S., Abdelmalek M.F., Caldwell S., Barb D., Kleiner D.E., Loomba R. (2023). AASLD Practice Guidance on the clinical assessment and management of nonalcoholic fatty liver disease. Hepatology.

[29] Arroyave-Ospina J.C., Wu Z., Geng Y., Moshage H. (2021). Role of Oxidative Stress in the Pathogenesis of Non-Alcoholic Fatty Liver Disease: Implications for Prevention and Therapy. Antioxidants.

[30] An J., Sohn J.H. (2023). Pharmacological advances in the treatment of nonalcoholic fatty liver diseases: Focused on global results of randomized controlled trials. Clin. Mol. Hepatol..

[31] Rosa A.C., Corsi D., Cavi N., Bruni N., Dosio F. (2021). Superoxide Dismutase Administration: A Review of Proposed Human Uses. Molecules.

[32] Sun Y.L., Bai T., Zhou L., Zhu R.T., Wang W.J., Liang R.P., Li J., Zhang C.X., Gou J.J. (2021). SOD3 deficiency induces liver fibrosis by promoting hepatic stellate cell activation and epithelial-mesenchymal transition. J. Cell. Physiol..

[33] Wang Y., Ding Y., Sun P., Zhang W., Xin Q., Wang N., Niu Y., Chen Y., Luo J., Lu J. (2022). Empagliflozin-Enhanced Antioxidant Defense Attenuates Lipotoxicity and Protects Hepatocytes by Promoting FoxO3a- and Nrf2-Mediated Nuclear Translocation via the CAMKK2/AMPK Pathway. Antioxidants.

[34] Barthel A., Schmoll D., Krüger K.D., Roth R.A., Joost H.G. (2002). Regulation of the forkhead transcription factor FKHR (FOXO1a) by glucose starvation and AICAR, an activator of AMP-activated protein kinase. Endocrinology.

[35] Pettinelli P., Videla L.A. (2011). Up-regulation of PPAR-gamma mRNA expression in the liver of obese patients: An additional reinforcing lipogenic mechanism to SREBP-1c induction. J. Clin. Endocrinol. Metab..

[36] Schadinger S.E., Bucher N.L., Schreiber B.M., Farmer S.R. (2005). PPARgamma2 regulates lipogenesis and lipid accumulation in steatotic hepatocytes. Am. J. Physiol. Endocrinol. Metab..

[37] Panasiuk A., Dzieciol J., Panasiuk B., Prokopowicz D. (2006). Expression of p53, Bax and Bcl-2 proteins in hepatocytes in non-alcoholic fatty liver disease. World J. Gastroenterol..

[38] Park H., Hasegawa G., Obayashi H., Fujinami A., Ohta M., Hara H., Adachi T., Tamaki S., Nakajima Y., Kimura F. (2006). Relationship between insulin resistance and inflammatory markers and anti-inflammatory effect of losartan in patients with type 2 diabetes and hypertension. Clin. Chim. Acta.

[39] Sasaki T., Abe Y., Takayama M., Adachi T., Okano H., Hirose N., Arai Y. (2021). Association among extracellular superoxide dismutase genotype, plasma concentration, and comorbidity in the very old and centenarians. Sci. Rep..

[40] Mohammedi K., Bellili-Munoz N., Marklund S.L., Driss F., Le Nagard H., Patente T.A., Fumeron F., Roussel R., Hadjadj S., Marre M. (2015). Plasma extracellular superoxide dismutase concentration, allelic variations in the SOD3 gene and risk of myocardial infarction and all-cause mortality in people with type 1 and type 2 diabetes. Cardiovasc. Diabetol..

[41] Sanyal A.J., Shankar S.S., Yates K.P., Bolognese J., Daly E., Dehn C.A., Neuschwander-Tetri B., Kowdley K., Vuppalanchi R., Behling C. (2023). Diagnostic performance of circulating biomarkers for non-alcoholic steatohepatitis. Nat. Med..

[42] Tincopa M.A., Loomba R. (2023). Non-invasive diagnosis and monitoring of non-alcoholic fatty liver disease and non-alcoholic steatohepatitis. Lancet Gastroenterol. Hepatol..

[43] Koruk M., Taysi S., Savas M.C., Yilmaz O., Akcay F., Karakok M. (2004). Oxidative stress and enzymatic antioxidant status in patients with nonalcoholic steatohepatitis. Ann. Clin. Lab. Sci..

[44] Kumar A., Sharma A., Duseja A., Das A., Dhiman R.K., Chawla Y.K., Kohli K.K., Bhansali A. (2013). Patients with Nonalcoholic Fatty Liver Disease (NAFLD) have Higher Oxidative Stress in Comparison to Chronic Viral Hepatitis. J. Clin. Exp. Hepatol..

[45] Yesilova Z., Yaman H., Oktenli C., Ozcan A., Uygun A., Cakir E., Sanisoglu S.Y., Erdil A., Ates Y., Aslan M. (2005). Systemic markers of lipid peroxidation and antioxidants in patients with nonalcoholic Fatty liver disease. Am. J. Gastroenterol..

[46] Yao N., He Y., Wu Y., Wang F., Tian Z. (2022). Prognostic value of plasma level of superoxide dismutase in HBV-related acute-on-chronic liver failure. BMC Gastroenterol..

[47] He Y., Wang F., Yao N., Wu Y., Zhao Y., Tian Z. (2022). Serum superoxide dismutase level is a potential biomarker of disease prognosis in patients with HEV-induced liver failure. BMC Gastroenterol..

[48] Tian Z., Yao N., Wu Y., Wang F., Zhao Y. (2022). Association between plasma level of superoxide dismutase and survival of patients with acute-on-chronic liver failure. BMC Gastroenterol..

